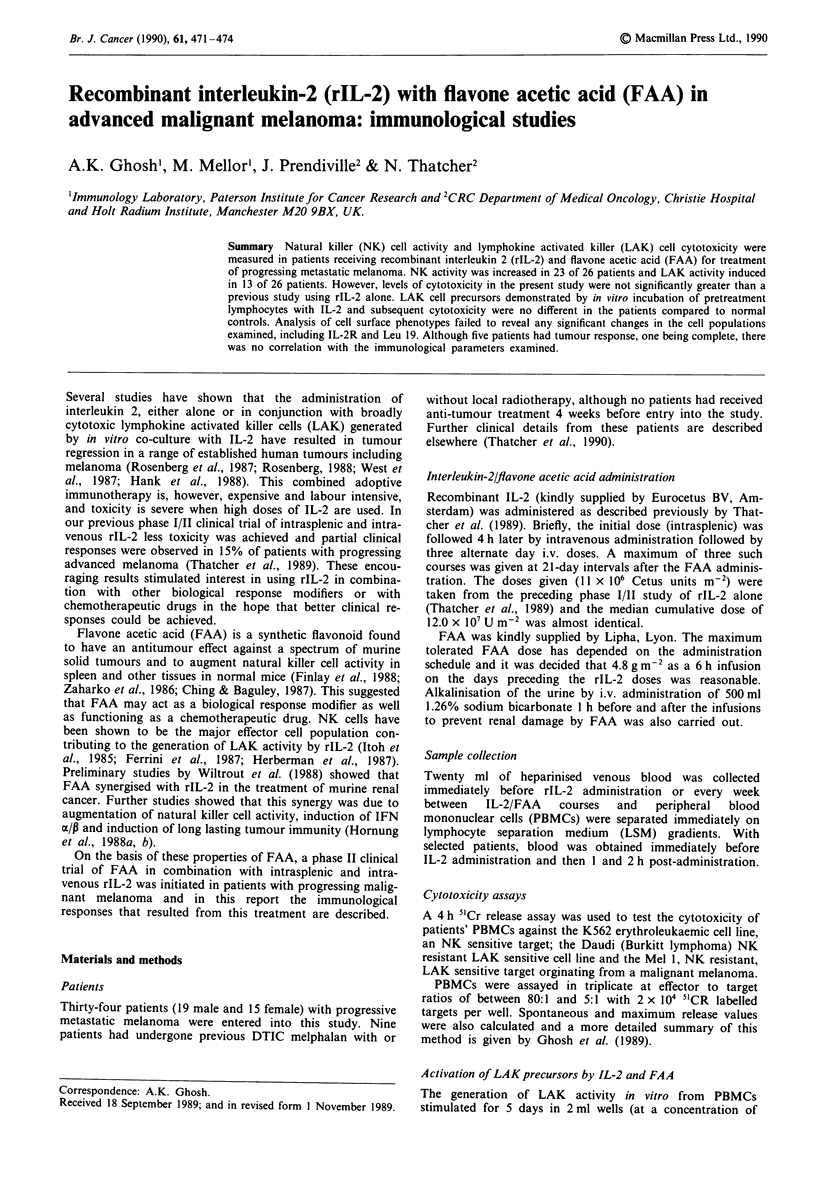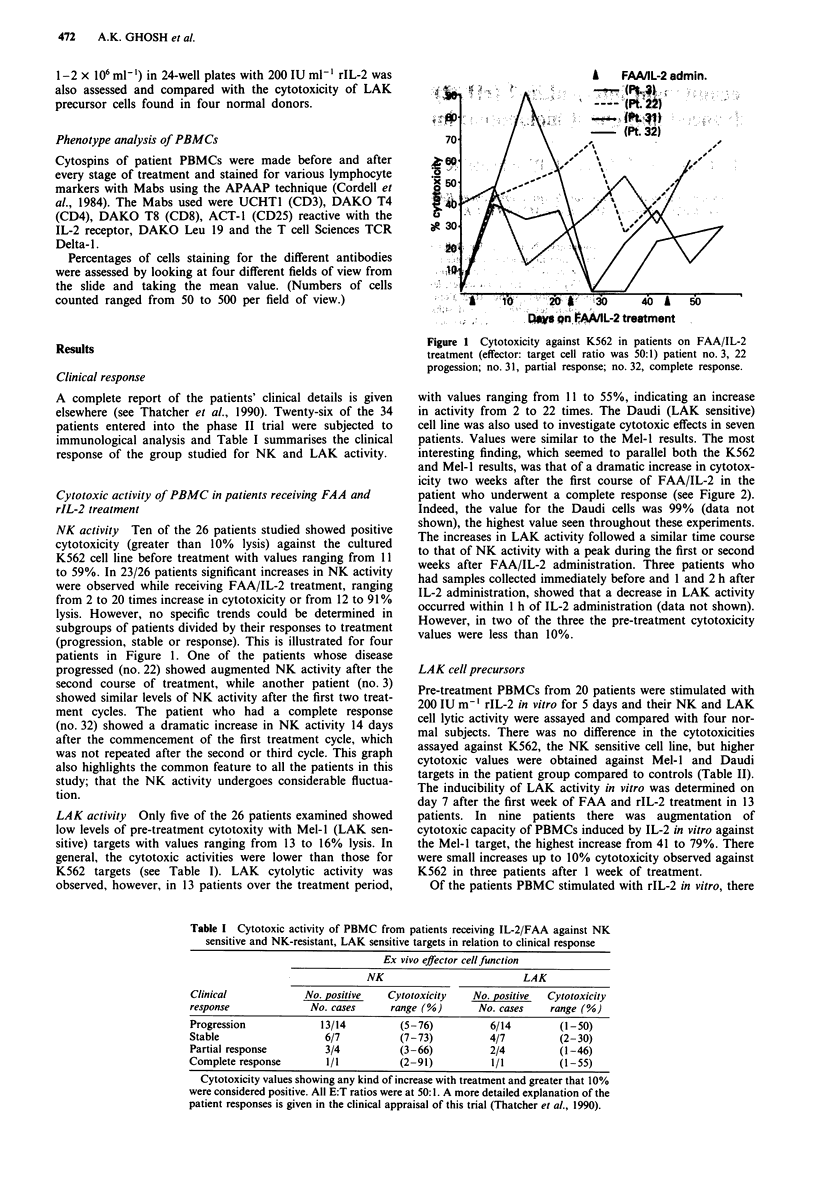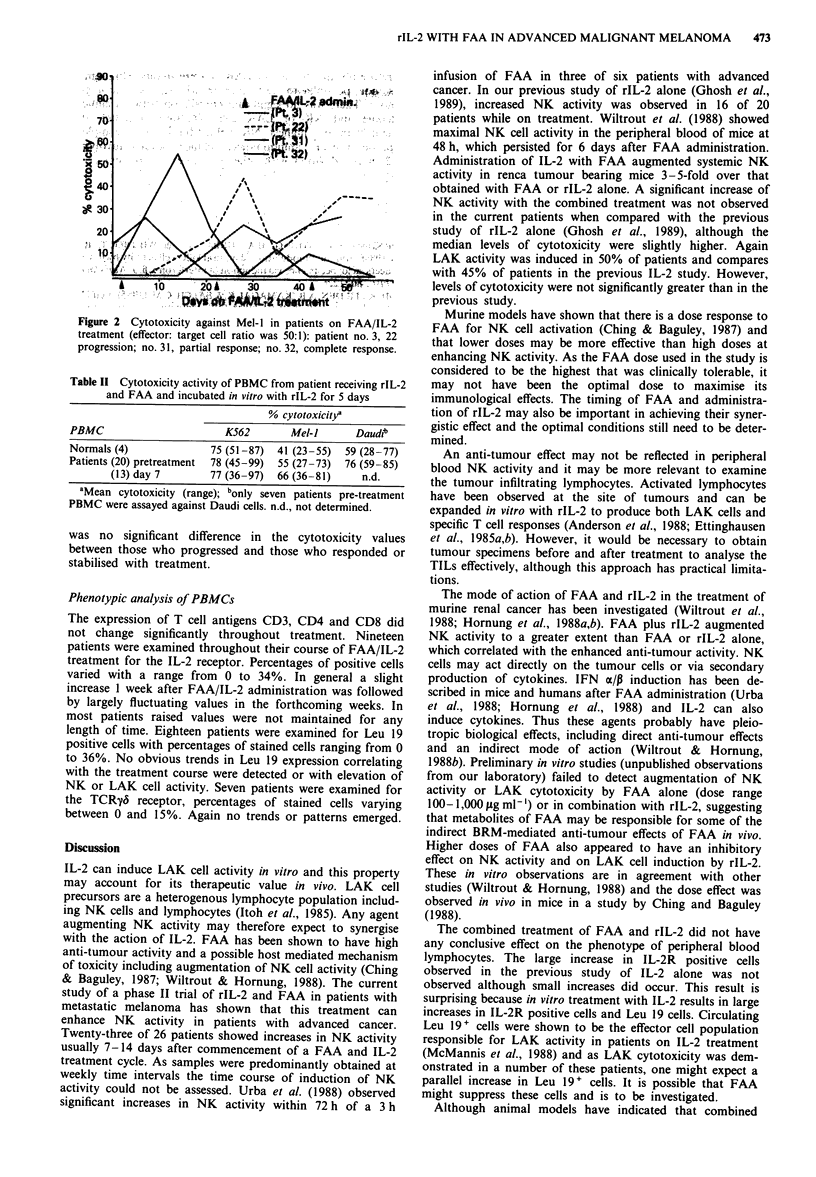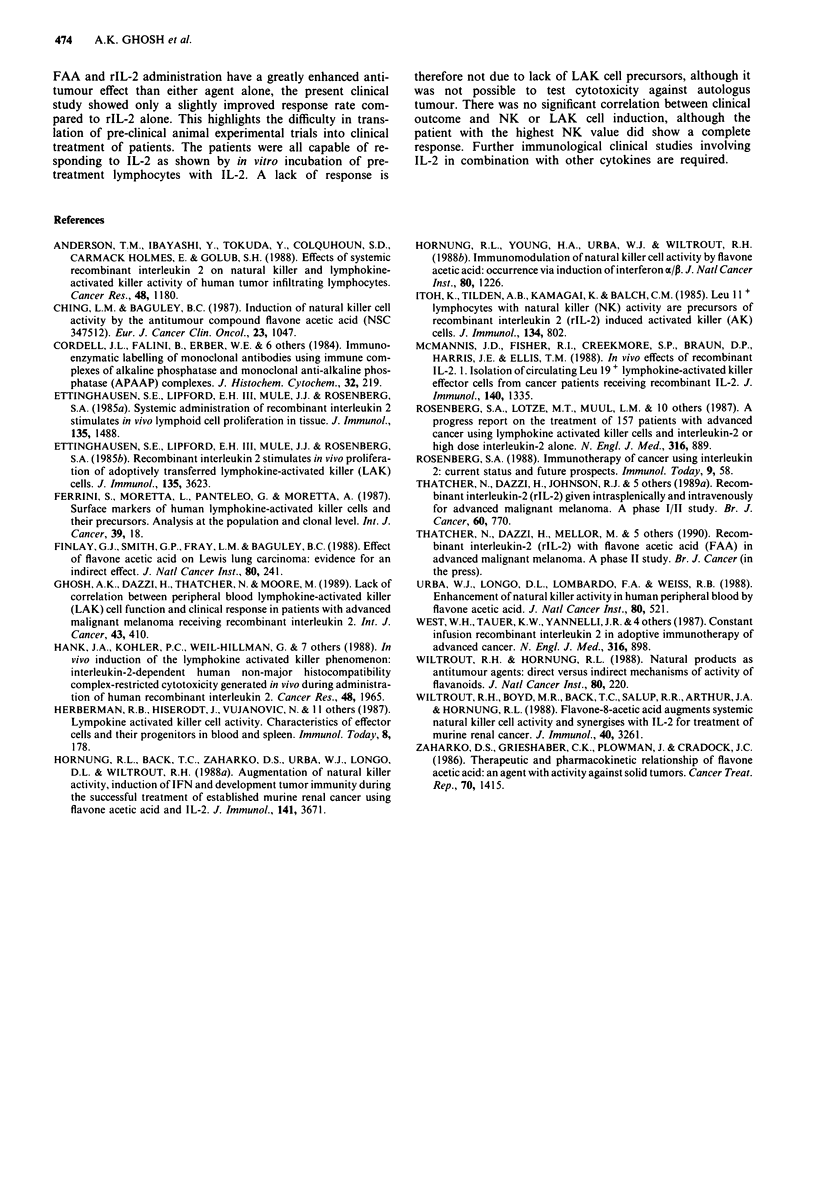# Recombinant interleukin-2 (rIL-2) with flavone acetic acid (FAA) in advanced malignant melanoma: immunological studies.

**DOI:** 10.1038/bjc.1990.104

**Published:** 1990-03

**Authors:** A. K. Ghosh, M. Mellor, J. Prendiville, N. Thatcher

**Affiliations:** Immunology Laboratory, Paterson Institute for Cancer Research, Manchester, UK.

## Abstract

Natural killer (NK) cell activity and lymphokine activated killer (LAK) cell cytotoxicity were measured in patients receiving recombinant interleukin 2 (rIL-2) and flavone acetic acid (FAA) for treatment of progressing metastatic melanoma. NK activity was increased in 23 of 26 patients and LAK activity induced in 13 of 26 patients. However, levels of cytotoxicity in the present study were not significantly greater than a previous study using rIL-2 alone. LAK cell precursors demonstrated by in vitro incubation of pretreatment lymphocytes with IL-2 and subsequent cytotoxicity were no different in the patients compared to normal controls. Analysis of cell surface phenotypes failed to reveal any significant changes in the cell populations examined, including IL-2R and Leu 19. Although five patients had tumour response, one being complete, there was no correlation with the immunological parameters examined.


Br. J. Cancer (1990), 61, 471-474 © Macmillan Press Ltd., 1990

### Recombinant interleukin-2 (rIL-2) with flavone acetic acid (FAA) in advanced malignant melanoma: immunological studies

A.K. Ghosh[1], M. Mellor[1], J. Prendiville[2] & N. Thatcher[2]

[1]*Immunology Laboratory, Paterson Institute for Cancer Research and* [2]*CRC Department of Medical Oncology, Christie Hospital and Holt Radium Institute, Manchester M20 9BX, UK.*

**Summary** Natural killer (NK) cell activity and lymphokine activated killer (LAK) cell cytotoxicity were measured in patients receiving recombinant interleukin 2 (rIL-2) and flavone acetic acid (FAA) for treatment of progressing metastatic melanoma. NK activity was increased in 23 of 26 patients and LAK activity induced in 13 of 26 patients. However, levels of cytotoxicity in the present study were not significantly greater than a previous study using rIL-2 alone. LAK cell precursors demonstrated by *in vitro* incubation of pretreatment lymphocytes with IL-2 and subsequent cytotoxicity were no different in the patients compared to normal controls. Analysis of cell surface phenotypes failed to reveal any significant changes in the cell populations examined, including IL-2R and Leu 19. Although five patients had tumour response, one being complete, there was no correlation with the immunological parameters examined.

Several studies have shown that the administration of interleukin 2, either alone or in conjunction with broadly cytotoxic lymphokine activated killer cells (LAK) generated by *in vitro* co-culture with IL-2 have resulted in tumour regression in a range of established human tumours including melanoma (Rosenberg *et al.*, 1987; Rosenberg, 1988; West *et al.*, 1987; Hank *et al.*, 1988). This combined adoptive immunotherapy is, however, expensive and labour intensive, and toxicity is severe when high doses of IL-2 are used. In our previous phase I/II clinical trial of intrasplenic and intravenous rIL-2 less toxicity was achieved and partial clinical responses were observed in 15% of patients with progressing advanced melanoma (Thatcher *et al.*, 1989). These encouraging results stimulated interest in using rIL-2 in combination with other biological response modifiers or with chemotherapeutic drugs in the hope that better clinical responses could be achieved.

Flavone acetic acid (FAA) is a synthetic flavonoid found to have an antitumour effect against a spectrum of murine solid tumours and to augment natural killer cell activity in spleen and other tissues in normal mice (Finlay *et al.*, 1988; Zaharko *et al.*, 1986; Ching & Baguley, 1987). This suggested that FAA may act as a biological response modifier as well as functioning as a chemotherapeutic drug. NK cells have been shown to be the major effector cell population contributing to the generation of LAK activity by rIL-2 (Itoh *et al.*, 1985; Ferrini *et al.*, 1987; Herberman *et al.*, 1987). Preliminary studies by Wiltrout *et al.* (1988) showed that FAA synergised with rIL-2 in the treatment of murine renal cancer. Further studies showed that this synergy was due to augmentation of natural killer cell activity, induction of IFN α/β and induction of long lasting tumour immunity (Hornung *et al.*, 1988*a*, *b*).

On the basis of these properties of FAA, a phase II clinical trial of FAA in combination with intrasplenic and intravenous rIL-2 was initiated in patients with progressing malignant melanoma and in this report the immunological responses that resulted from this treatment are described.

### Materials and methods

### *Patients*

Thirty-four patients (19 male and 15 female) with progressive metastatic melanoma were entered into this study. Nine patients had undergone previous DTIC melphalan with or without local radiotherapy, although no patients had received anti-tumour treatment 4 weeks before entry into the study. Further clinical details from these patients are described elsewhere (Thatcher *et al.*, 1990).

### *Interleukin-2/flavone acetic acid administration*

Recombinant IL-2 (kindly supplied by Eurocetus BV, Amsterdam) was administered as described previously by Thatcher *et al.* (1989). Briefly, the initial dose (intrasplenic) was followed 4 h later by intravenous administration followed by three alternate day i.v. doses. A maximum of three such courses was given at 21-day intervals after the FAA administration. The doses given ($11 \times 10^6$ Cetus units $m^{-2}$) were taken from the preceding phase I/II study of rIL-2 alone (Thatcher *et al.*, 1989) and the median cumulative dose of $12.0 \times 10^7$ U $m^{-2}$ was almost identical.

FAA was kindly supplied by Lipha, Lyon. The maximum tolerated FAA dose has depended on the administration schedule and it was decided that 4.8 g $m^{-2}$ as a 6 h infusion on the days preceding the rIL-2 doses was reasonable. Alkalinisation of the urine by i.v. administration of 500 ml 1.26% sodium bicarbonate 1 h before and after the infusions to prevent renal damage by FAA was also carried out.

### *Sample collection*

Twenty ml of heparinised venous blood was collected immediately before rIL-2 administration or every week between IL-2/FAA courses and peripheral blood mononuclear cells (PBMCs) were separated immediately on lymphocyte separation medium (LSM) gradients. With selected patients, blood was obtained immediately before IL-2 administration and then 1 and 2 h post-administration.

### *Cytotoxicity assays*

A 4 h $^{51}Cr$ release assay was used to test the cytotoxicity of patients' PBMCs against the K562 erythroleukaemic cell line, an NK sensitive target; the Daudi (Burkitt lymphoma) NK resistant LAK sensitive cell line and the Mel 1, NK resistant, LAK sensitive target orginating from a malignant melanoma.

PBMCs were assayed in triplicate at effector to target ratios of between 80:1 and 5:1 with $2 \times 10^4$ $^{51}CR$ labelled targets per well. Spontaneous and maximum release values were also calculated and a more detailed summary of this method is given by Ghosh *et al.* (1989).

### *Activation of LAK precursors by IL-2 and FAA*

The generation of LAK activity *in vitro* from PBMCs stimulated for 5 days in 2 ml wells (at a concentration of

Correspondence: A.K. Ghosh.
Received 18 September 1989; and in revised form 1 November 1989.

1–2 × $10^6$ $ml^{-1}$) in 24-well plates with 200 IU $ml^{-1}$ rIL-2 was also assessed and compared with the cytotoxicity of LAK precursor cells found in four normal donors.

*Phenotype analysis of PBMCs*

Cytospins of patient PBMCs were made before and after every stage of treatment and stained for various lymphocyte markers with Mabs using the APAAP technique (Cordell *et al.*, 1984). The Mabs used were UCHT1 (CD3), DAKO T4 (CD4), DAKO T8 (CD8), ACT-1 (CD25) reactive with the IL-2 receptor, DAKO Leu 19 and the T cell Sciences TCR Delta-1.

Percentages of cells staining for the different antibodies were assessed by looking at four different fields of view from the slide and taking the mean value. (Numbers of cells counted ranged from 50 to 500 per field of view.)

### Results

*Clinical response*

A complete report of the patients' clinical details is given elsewhere (see Thatcher *et al.*, 1990). Twenty-six of the 34 patients entered into the phase II trial were subjected to immunological analysis and Table I summarises the clinical response of the group studied for NK and LAK activity.

*Cytotoxic activity of PBMC in patients receiving FAA and rIL-2 treatment*

*NK activity* Ten of the 26 patients studied showed positive cytotoxicity (greater than 10% lysis) against the cultured K562 cell line before treatment with values ranging from 11 to 59%. In 23/26 patients significant increases in NK activity were observed while receiving FAA/IL-2 treatment, ranging from 2 to 20 times increase in cytotoxicity or from 12 to 91% lysis. However, no specific trends could be determined in subgroups of patients divided by their responses to treatment (progression, stable or response). This is illustrated for four patients in Figure 1. One of the patients whose disease progressed (no. 22) showed augmented NK activity after the second course of treatment, while another patient (no. 3) showed similar levels of NK activity after the first two treatment cycles. The patient who had a complete response (no. 32) showed a dramatic increase in NK activity 14 days after the commencement of the first treatment cycle, which was not repeated after the second or third cycle. This graph also highlights the common feature to all the patients in this study; that the NK activity undergoes considerable fluctuation.

*LAK activity* Only five of the 26 patients examined showed low levels of pre-treatment cytotoxity with Mel-1 (LAK sensitive) targets with values ranging from 13 to 16% lysis. In general, the cytotoxic activities were lower than those for K562 targets (see Table I). LAK cytolytic activity was observed, however, in 13 patients over the treatment period, with values ranging from 11 to 55%, indicating an increase in activity from 2 to 22 times. The Daudi (LAK sensitive) cell line was also used to investigate cytotoxic effects in seven patients. Values were similar to the Mel-1 results. The most interesting finding, which seemed to parallel both the K562 and Mel-1 results, was that of a dramatic increase in cytotoxicity two weeks after the first course of FAA/IL-2 in the patient who underwent a complete response (see Figure 2). Indeed, the value for the Daudi cells was 99% (data not shown), the highest value seen throughout these experiments. The increases in LAK activity followed a similar time course to that of NK activity with a peak during the first or second weeks after FAA/IL-2 administration. Three patients who had samples collected immediately before and 1 and 2 h after IL-2 administration, showed that a decrease in LAK activity occurred within 1 h of IL-2 administration (data not shown). However, in two of the three the pre-treatment cytotoxicity values were less than 10%.

**Figure 1** Cytotoxicity against K562 in patients on FAA/IL-2 treatment (effector: target cell ratio was 50:1) patient no. 3, 22 progession; no. 31, partial response; no. 32, complete response.

*LAK cell precursors*

Pre-treatment PBMCs from 20 patients were stimulated with 200 IU $m^{-1}$ rIL-2 *in vitro* for 5 days and their NK and LAK cell lytic activity were assayed and compared with four normal subjects. There was no difference in the cytotoxicities assayed against K562, the NK sensitive cell line, but higher cytotoxic values were obtained against Mel-1 and Daudi targets in the patient group compared to controls (Table II). The inducibility of LAK activity *in vitro* was determined on day 7 after the first week of FAA and rIL-2 treatment in 13 patients. In nine patients there was augmentation of cytotoxic capacity of PBMCs induced by IL-2 *in vitro* against the Mel-1 target, the highest increase from 41 to 79%. There were small increases up to 10% cytotoxicity observed against K562 in three patients after 1 week of treatment.

Of the patients PBMC stimulated with rIL-2 *in vitro*, there

**Table I** Cytotoxic activity of PBMC from patients receiving IL-2/FAA against NK sensitive and NK-resistant, LAK sensitive targets in relation to clinical response

| | *Ex vivo effector cell function* | | | |
|---|---|---|---|---|
| | *NK* | | *LAK* | |
| *Clinical response* | *No. positive / No. cases* | *Cytotoxicity range (%)* | *No. positive / No. cases* | *Cytotoxicity range (%)* |
| Progression | 13/14 | (5–76) | 6/14 | (1–50) |
| Stable | 6/7 | (7–73) | 4/7 | (2–30) |
| Partial response | 3/4 | (3–66) | 2/4 | (1–46) |
| Complete response | 1/1 | (2–91) | 1/1 | (1–55) |

Cytotoxicity values showing any kind of increase with treatment and greater that 10% were considered positive. All E:T ratios were at 50:1. A more detailed explanation of the patient responses is given in the clinical appraisal of this trial (Thatcher *et al.*, 1990).

**Figure 2** Cytotoxicity against Mel-1 in patients on FAA/IL-2 treatment (effector: target cell ratio was 50:1): patient no. 3, 22 progression; no. 31, partial response; no. 32, complete response.

**Table II** Cytotoxicity activity of PBMC from patient receiving rIL-2 and FAA and incubated *in vitro* with rIL-2 for 5 days

| *PBMC* | % cytotoxicity[a] *K562* | *Mel-1* | *Daudi*[b] |
|---|---|---|---|
| Normals (4) | 75 (51–87) | 41 (23–55) | 59 (28–77) |
| Patients (20) pretreatment | 78 (45–99) | 55 (27–73) | 76 (59–85) |
| (13) day 7 | 77 (36–97) | 66 (36–81) | n.d. |

[a]Mean cytotoxicity (range); [b]only seven patients pre-treatment PBMC were assayed against Daudi cells. n.d., not determined.

was no significant difference in the cytotoxicity values between those who progressed and those who responded or stabilised with treatment.

*Phenotypic analysis of PBMCs*

The expression of T cell antigens CD3, CD4 and CD8 did not change significantly throughout treatment. Nineteen patients were examined throughout their course of FAA/IL-2 treatment for the IL-2 receptor. Percentages of positive cells varied with a range from 0 to 34%. In general a slight increase 1 week after FAA/IL-2 administration was followed by largely fluctuating values in the forthcoming weeks. In most patients raised values were not maintained for any length of time. Eighteen patients were examined for Leu 19 positive cells with percentages of stained cells ranging from 0 to 36%. No obvious trends in Leu 19 expression correlating with the treatment course were detected or with elevation of NK or LAK cell activity. Seven patients were examined for the TCR$\gamma\delta$ receptor, percentages of stained cells varying between 0 and 15%. Again no trends or patterns emerged.

**Discussion**

IL-2 can induce LAK cell activity *in vitro* and this property may account for its therapeutic value *in vivo*. LAK cell precursors are a heterogenous lymphocyte population including NK cells and lymphocytes (Itoh *et al*., 1985). Any agent augmenting NK activity may therefore expect to synergise with the action of IL-2. FAA has been shown to have high anti-tumour activity and a possible host mediated mechanism of toxicity including augmentation of NK cell activity (Ching & Baguley, 1987; Wiltrout & Hornung, 1988). The current study of a phase II trial of rIL-2 and FAA in patients with metastatic melanoma has shown that this treatment can enhance NK activity in patients with advanced cancer. Twenty-three of 26 patients showed increases in NK activity usually 7–14 days after commencement of a FAA and IL-2 treatment cycle. As samples were predominantly obtained at weekly time intervals the time course of induction of NK activity could not be assessed. Urba *et al*. (1988) observed significant increases in NK activity within 72 h of a 3 h infusion of FAA in three of six patients with advanced cancer. In our previous study of rIL-2 alone (Ghosh *et al*., 1989), increased NK activity was observed in 16 of 20 patients while on treatment. Wiltrout *et al*. (1988) showed maximal NK cell activity in the peripheral blood of mice at 48 h, which persisted for 6 days after FAA administration. Administration of IL-2 with FAA augmented systemic NK activity in renca tumour bearing mice 3–5-fold over that obtained with FAA or rIL-2 alone. A significant increase of NK activity with the combined treatment was not observed in the current patients when compared with the previous study of rIL-2 alone (Ghosh *et al*., 1989), although the median levels of cytotoxicity were slightly higher. Again LAK activity was induced in 50% of patients and compares with 45% of patients in the previous IL-2 study. However, levels of cytotoxicity were not significantly greater than in the previous study.

Murine models have shown that there is a dose response to FAA for NK cell activation (Ching & Baguley, 1987) and that lower doses may be more effective than high doses at enhancing NK activity. As the FAA dose used in the study is considered to be the highest that was clinically tolerable, it may not have been the optimal dose to maximise its immunological effects. The timing of FAA and administration of rIL-2 may also be important in achieving their synergistic effect and the optimal conditions still need to be determined.

An anti-tumour effect may not be reflected in peripheral blood NK activity and it may be more relevant to examine the tumour infiltrating lymphocytes. Activated lymphocytes have been observed at the site of tumours and can be expanded *in vitro* with rIL-2 to produce both LAK cells and specific T cell responses (Anderson *et al*., 1988; Ettinghausen *et al*., 1985*a*,*b*). However, it would be necessary to obtain tumour specimens before and after treatment to analyse the TILs effectively, although this approach has practical limitations.

The mode of action of FAA and rIL-2 in the treatment of murine renal cancer has been investigated (Wiltrout *et al*., 1988; Hornung *et al*., 1988*a*,*b*). FAA plus rIL-2 augmented NK activity to a greater extent than FAA or rIL-2 alone, which correlated with the enhanced anti-tumour activity. NK cells may act directly on the tumour cells or via secondary production of cytokines. IFN $\alpha/\beta$ induction has been described in mice and humans after FAA administration (Urba *et al*., 1988; Hornung *et al*., 1988) and IL-2 can also induce cytokines. Thus these agents probably have pleiotropic biological effects, including direct anti-tumour effects and an indirect mode of action (Wiltrout & Hornung, 1988*b*). Preliminary *in vitro* studies (unpublished observations from our laboratory) failed to detect augmentation of NK activity or LAK cytotoxicity by FAA alone (dose range 100–1,000 $\mu g\ ml^{-1}$) or in combination with rIL-2, suggesting that metabolites of FAA may be responsible for some of the indirect BRM-mediated anti-tumour effects of FAA *in vivo*. Higher doses of FAA also appeared to have an inhibitory effect on NK activity and on LAK cell induction by rIL-2. These *in vitro* observations are in agreement with other studies (Wiltrout & Hornung, 1988) and the dose effect was observed *in vivo* in mice in a study by Ching and Baguley (1988).

The combined treatment of FAA and rIL-2 did not have any conclusive effect on the phenotype of peripheral blood lymphocytes. The large increase in IL-2R positive cells observed in the previous study of IL-2 alone was not observed although small increases did occur. This result is surprising because *in vitro* treatment with IL-2 results in large increases in IL-2R positive cells and Leu 19 cells. Circulating Leu $19^+$ cells were shown to be the effector cell population responsible for LAK activity in patients on IL-2 treatment (McMannis *et al*., 1988) and as LAK cytotoxicity was demonstrated in a number of these patients, one might expect a parallel increase in Leu $19^+$ cells. It is possible that FAA might suppress these cells and is to be investigated.

Although animal models have indicated that combined

FAA and rIL-2 administration have a greatly enhanced antitumour effect than either agent alone, the present clinical study showed only a slightly improved response rate compared to rIL-2 alone. This highlights the difficulty in translation of pre-clinical animal experimental trials into clinical treatment of patients. The patients were all capable of responding to IL-2 as shown by *in vitro* incubation of pretreatment lymphocytes with IL-2. A lack of response is therefore not due to lack of LAK cell precursors, although it was not possible to test cytotoxicity against autologus tumour. There was no significant correlation between clinical outcome and NK or LAK cell induction, although the patient with the highest NK value did show a complete response. Further immunological clinical studies involving IL-2 in combination with other cytokines are required.

### References

ANDERSON, T.M., IBAYASHI, Y., TOKUDA, Y., COLQUHOUN, S.D., CARMACK HOLMES, E. & GOLUB, S.H. (1988). Effects of systemic recombinant interleukin 2 on natural killer and lymphokine-activated killer activity of human tumor infiltrating lymphocytes. *Cancer Res.*, **48**, 1180.

CHING, L.M. & BAGULEY, B.C. (1987). Induction of natural killer cell activity by the antitumour compound flavone acetic acid (NSC 347512). *Eur. J. Cancer Clin. Oncol.*, **23**, 1047.

CORDELL, J.L., FALINI, B., ERBER, W.E. & 6 others (1984). Immunoenzymatic labelling of monoclonal antibodies using immune complexes of alkaline phosphatase and monoclonal anti-alkaline phosphatase (APAAP) complexes. *J. Histochem. Cytochem.*, **32**, 219.

ETTINGHAUSEN, S.E., LIPFORD, E.H. III, MULE, J.J. & ROSENBERG, S.A. (1985*a*). Systemic administration of recombinant interleukin 2 stimulates *in vivo* lymphoid cell proliferation in tissue. *J. Immunol.*, **135**, 1488.

ETTINGHAUSEN, S.E., LIPFORD, E.H. III, MULE, J.J. & ROSENBERG, S.A. (1985*b*). Recombinant interleukin 2 stimulates *in vivo* proliferation of adoptively transferred lymphokine-activated killer (LAK) cells. *J. Immunol.*, **135**, 3623.

FERRINI, S., MORETTA, L., PANTELEO, G. & MORETTA, A. (1987). Surface markers of human lymphokine-activated killer cells and their precursors. Analysis at the population and clonal level. *Int. J. Cancer*, **39**, 18.

FINLAY, G.J., SMITH, G.P., FRAY, L.M. & BAGULEY, B.C. (1988). Effect of flavone acetic acid on Lewis lung carcinoma: evidence for an indirect effect. *J. Natl Cancer Inst.*, **80**, 241.

GHOSH, A.K., DAZZI, H., THATCHER, N. & MOORE, M. (1989). Lack of correlation between peripheral blood lymphokine-activated killer (LAK) cell function and clinical response in patients with advanced malignant melanoma receiving recombinant interleukin 2. *Int. J. Cancer*, **43**, 410.

HANK, J.A., KOHLER, P.C., WEIL-HILLMAN, G. & 7 others (1988). *In vivo* induction of the lymphokine activated killer phenomenon: interleukin-2-dependent human non-major histocompatibility complex-restricted cytotoxicity generated *in vivo* during administration of human recombinant interleukin 2. *Cancer Res.*, **48**, 1965.

HERBERMAN, R.B., HISERODT, J., VUJANOVIC, N. & 11 others (1987). Lympokine activated killer cell activity. Characteristics of effector cells and their progenitors in blood and spleen. *Immunol. Today*, **8**, 178.

HORNUNG, R.L., BACK, T.C., ZAHARKO, D.S., URBA, W.J., LONGO, D.L. & WILTROUT, R.H. (1988*a*). Augmentation of natural killer activity, induction of IFN and development tumor immunity during the successful treatment of established murine renal cancer using flavone acetic acid and IL-2. *J. Immunol.*, **141**, 3671.

HORNUNG, R.L., YOUNG, H.A., URBA, W.J. & WILTROUT, R.H. (1988*b*). Immunomodulation of natural killer cell activity by flavone acetic acid: occurrence via induction of interferon $\alpha/\beta$. *J. Natl Cancer Inst.*, **80**, 1226.

ITOH, K., TILDEN, A.B., KAMAGAI, K. & BALCH, C.M. (1985). Leu 11$^+$ lymphocytes with natural killer (NK) activity are precursors of recombinant interleukin 2 (rIL-2) induced activated killer (AK) cells. *J. Immunol.*, **134**, 802.

McMANNIS, J.D., FISHER, R.I., CREEKMORE, S.P., BRAUN, D.P., HARRIS, J.E. & ELLIS, T.M. (1988). *In vivo* effects of recombinant IL-2. 1. Isolation of circulating Leu 19$^+$ lymphokine-activated killer effector cells from cancer patients receiving recombinant IL-2. *J. Immunol.*, **140**, 1335.

ROSENBERG, S.A., LOTZE, M.T., MUUL, L.M. & 10 others (1987). A progress report on the treatment of 157 patients with advanced cancer using lymphokine activated killer cells and interleukin-2 or high dose interleukin-2 alone. *N. Engl. J. Med.*, **316**, 889.

ROSENBERG, S.A. (1988). Immunotherapy of cancer using interleukin 2: current status and future prospects. *Immunol. Today*, **9**, 58.

THATCHER, N., DAZZI, H., JOHNSON, R.J. & 5 others (1989*a*). Recombinant interleukin-2 (rIL-2) given intrasplenically and intravenously for advanced malignant melanoma. A phase I/II study. *Br. J. Cancer*, **60**, 770.

THATCHER, N., DAZZI, H., MELLOR, M. & 5 others (1990). Recombinant interleukin-2 (rIL-2) with flavone acetic acid (FAA) in advanced malignant melanoma. A phase II study. *Br. J. Cancer* (in the press).

URBA, W.J., LONGO, D.L., LOMBARDO, F.A. & WEISS, R.B. (1988). Enhancement of natural killer activity in human peripheral blood by flavone acetic acid. *J. Natl Cancer Inst.*, **80**, 521.

WEST, W.H., TAUER, K.W., YANNELLI, J.R. & 4 others (1987). Constant infusion recombinant interleukin 2 in adoptive immunotherapy of advanced cancer. *N. Engl. J. Med.*, **316**, 898.

WILTROUT, R.H. & HORNUNG, R.L. (1988). Natural products as antitumour agents: direct versus indirect mechanisms of activity of flavanoids. *J. Natl Cancer Inst.*, **80**, 220.

WILTROUT, R.H., BOYD, M.R., BACK, T.C., SALUP, R.R., ARTHUR, J.A. & HORNUNG, R.L. (1988). Flavone-8-acetic acid augments systemic natural killer cell activity and synergises with IL-2 for treatment of murine renal cancer. *J. Immunol.*, **40**, 3261.

ZAHARKO, D.S., GRIESHABER, C.K., PLOWMAN, J. & CRADOCK, J.C. (1986). Therapeutic and pharmacokinetic relationship of flavone acetic acid: an agent with activity against solid tumors. *Cancer Treat. Rep.*, **70**, 1415.